# COVID-19 in pulmonary critically ill patients: metagenomic identification of fungi and characterization of pathogenic microorganisms

**DOI:** 10.3389/fcimb.2023.1220012

**Published:** 2024-02-20

**Authors:** Changjun Huang, Siyuan Chang, Rui Ma, Yishu Shang, Yuexia Li, Yun Wang, Min Feng, Wenzhi Guo

**Affiliations:** ^1^ Department of Hepatobiliary Surgery, The First Affiliated Hospital of Zhengzhou University, Zhengzhou, China; ^2^ Department of Surgical Intensive Care Unit, The First Affiliated Hospital of Zhengzhou University, Zhengzhou, China

**Keywords:** COVID-19, SARS-CoV-2, aspergillosis, mNGS, CAPA

## Abstract

**Background:**

Fungal co-infection is prevalent in critically ill patients with COVID-19. The conventional approach applied to fungal identification has relatively low sensitivity and is time-consuming. The metagenomic next-generation sequencing (mNGS) technology can simultaneously detect a variety of microorganisms, and is increasingly being used for the rapid detection and diagnosis of pathogens.

**Methods:**

In this single-center retrospective study, we described the clinical presentation and outcomes of COVID-19 and mNGS positive for fungi in pulmonary critically ill patients during the outbreak of Omicron infection from December 2022 to January 2023.

**Results:**

Among 43 COVID-19 patients with acute respiratory distress syndrome (ARDS) on a single intensive care unit (ICU), 10 were reported to be fungal positive using the mNGS test. The number of pathogenic microorganisms detected by mNGS was significantly higher than that via traditional methods, especially in the detection of fungi and viruses. *Aspergillus* infection was dominant, and most of these patients also had concurrent bacterial or viral infections. Probable or possible COVID-19-associated pulmonary aspergillosis (CAPA) was diagnosed in all 10 patients, and the prognosis was poor.

**Conclusion:**

Patients with COVID-19 may be at increased risk of developing fungal infections as well as concurrent bacterial or viral infections, and mNGS can be a powerful tool in identifying these infections. Clinicians should be aware of the increased risk of fungal infections in COVID-19 patients, particularly those who have underlying immunocompromising conditions, and should monitor for early signs of infection.

## 1 Introduction

The pandemic of an infectious disease named coronavirus disease 2019 (COVID-19) caused by severe acute respiratory syndrome coronavirus 2 (SARS-CoV-2) has become a worldwide threat (Shereen et al., 2020; Hu et al., 2021). COVID-19 infection has rapidly spread over China since December 2022 despite restrictive control measures by the government (The, 2023). As the infection continued to spread, severe pneumonia patients infected with SARS-CoV-2 requiring intensive care unit (ICU) admission gradually emerged.

The prevalence of fungal co-infection was reported in critically ill patients with COVID-19 and the mortality rate remains high (Bhatt et al., 2021; Seyedjavadi et al., 2022). A pre-Omicron outbreak retrospective observational study conducted in China revealed that 5.8% (3/52) of critically ill patients exhibited fungal co-infections (Yang et al., 2020). The identified infectious fungi included *Aspergillus flavus*, *Aspergillus fumigatus*, and *Candida albicans*. As far as our knowledge extends, following the emergence of the Omicron variant in China, substantial data encompassing a large sample size to characterize co-existing fungal infections in COVID-19 cases are currently lacking. Therefore, further clinical evidence in relation to the COVID-19 and fungal co-infection in critically ill patients is necessary. Rapid identification of pulmonary fungal infections facilitates the well-timed antifungal therapy. Despite the fact that microscopy and culture are the conventional approach applied to fungal identification, both methods have a relatively low sensitivity, and culture is a time-consuming process because of the long fungal growth time (Li, 2022). Histopathological diagnosis is still the gold standard for identifying invasive fungal infections (IFIs), despite it being a lengthy process (Mendonca et al., 2022). Immunological recognition of serum galactomannan and β-d-glucan antigen has significant auxiliary diagnostic efficacy, but there are many cofounding factors (Li, 2022). The metagenomic next-generation sequencing (mNGS) technology is a high-throughput DNA or RNA sequencing method that can simultaneously detect a variety of microorganisms, including bacteria, viruses, and fungi, and is widely used for the rapid detection and diagnosis of pathogens (Gu et al., 2021). Although mNGS has disadvantages such as its high cost, susceptibility to exogenous microbial contamination, and reduced efficiency in detecting thick-walled fungi, research has showcased its superior sensitivity and specificity in diagnosing invasive fungal diseases compared to traditional culture and histopathological methods (El-Kamand et al., 2019; Song et al., 2021; Wang et al., 2022). In certain circumstances, mNGS has a superior positive detection efficiency and can determine fungal pathogens that are difficult to diagnose via conventional diagnostic approaches (Zheng et al., 2021; Wu et al., 2023).

In this single-center retrospective study, we described the clinical presentation and outcomes of COVID-19 and mNGS positive for fungi in pulmonary critically ill patients during the outbreak of Omicron infection from December 2022 to January 2023.

## 2 Patients and methods

### 2.1 Study cohort

This is a retrospective single-center cohort study in China. Patients who were admitted to the ICU at the First Affiliated Hospital of Zhengzhou University between 29 December 2022 and 23 January 2023 were reviewed. All adult patients (aged > 18 years) who were diagnosed with COVID-19 and acute respiratory distress syndrome (ARDS), and who had undergone mNGS testing at least once, were screened. Furthermore, patients positive for fungi via mNGS testing were included in our study.

### 2.2 Data collection

Demographic, epidemiological, clinical, laboratory, treatment, and outcome data were retrieved from electronic medical records utilizing a standardized data collection form. All data were examined by two physicians (CH and SC), and a third researcher (YW) resolved contradictions between the two reviewers, if any.

### 2.3 Laboratory procedures

Methods for laboratory identification of SARS-CoV-2 infection have been described previously (Huang et al., 2020a). Briefly, the clinical laboratory of the First Affiliated Hospital of Zhengzhou University was responsible for SARS-CoV-2 detection in respiratory specimens using real-time RT-PCR or the antigen rapid detection test. SARS-CoV-2 and other pathogen detection in bronchoalveolar lavage fluid (BALF) or peripheral blood (PB) by mNGS was performed by the clinical laboratory of the First Affiliated Hospital of Zhengzhou University or Hangzhou Matridx Biotechnology Co., Ltd., Zhejiang, China. Initial data from the mNGS upon admission to the ICU were collected for analysis if the patient had multiple mNGS tests.

Results of the first examination after admission to ICU of routine blood examinations, procalcitonin (PCT), C-reactive protein (CRP), interleukin-6 (IL-6), N-terminal pro-brain natriuretic peptide (BNP), creatinine (Cr), urea, prothrombin time (PT), activated partial thromboplastin time (APTT), d-dimer, glucose (Glu), alanine aminotransferase (ALT), total bilirubin (TBIL), lactic acid (Lac), high-sensitivity cardiac troponin T (TNT), high-sensitivity cardiac troponin I (TNI), creatine kinase-MB (CK-MB), lymphocyte immunoassay, galactomannan, β-D-glucan, and microbial culture of PB, BALF, and sputum were collected for further analysis. Variables might be unavailable in some circumstances. Chest x-ray or CT scan was also performed for all patients. Frequency of examinations was determined by the managing physician. SOFA score, CURB-65 score, and APACHE II score were collected upon admission to the ICU.

### 2.4 mNGS detection protocol

*Nucleic acid extraction, library preparation, and sequencing:* For the BALF, cell walls of microbes were broken by vortex with glass beads. For the blood, the plasma was separated. DNA was extracted using the TIANamp micro DNA kit (DP316; Tiangen Biotech, Beijing, China) following the manufacturer’s operation manual. Total RNA was extracted with a QIAamp^®^ Viral RNA Kit (Qiagen) and ribosomal RNA was removed using a Ribo-Zero rRNA Removal Kit (Illumina). cDNA was generated using reverse transcriptase, random hexamer primers, and dNTPs (Thermo Fisher). Libraries were constructed for the DNA and cDNA samples using a Nextera XT DNA Library Prep Kit (Illumina, San Diego, CA). The library was purified and the fragments were selected by the magnetic beads. The library was quality assessed by the Qubit dsDNA HS Assay kit followed by the High Sensitivity DNA kit (Agilent) on an Agilent 2100 Bioanalyzer. The size of the qualified library was 300 to 500 bp without adapters and PCR dimers, and the concentration of the library was greater than 1 ng/mL. Library pools were then loaded onto an Illumina Nextseq 500CN sequencer for 75 cycles of single-end sequencing to generate approximately 20 million reads for each library. The internal control, named UMSI (unique molecular spiked-in), was added to the sample before the DNA extraction. The sequence of UMSI varied in different samples. Each mNGS assay run included an external negative control that ran in parallel with clinical samples. During analysis, the contamination between samples can be found if the UMSI sequence was the same or the reads of some pathogens in the external control were very high.

*Bioinformatics analyses:* High-quality sequencing data were generated by removing low-quality and short (length < 40 bp) reads, followed by computational subtraction of human host sequences mapped to the human reference genome (hg38 and YH sequences) using Burrows–Wheeler Alignment. After the removal of low-complexity reads, the remaining data were classified by simultaneously aligning them to four microbial genome databases, namely, viruses, bacteria, fungi, and parasites. The classification reference databases were downloaded and optimized from public databases such as NCBI, EBI, or Genbank. In the end, the multi-parameters of species in microbial genome databases were calculated and exported, and professionals with microbiology and clinical backgrounds interpreted the results.

### 2.5 Definitions

ARDS was diagnosed in accordance with the Berlin definition (Force et al., 2012). For the diagnosis of COVID-19-associated invasive pulmonary aspergillosis (CAPA), the modified ECMM/ISHAM 2020 consensus criteria were adopted (Fortun et al., 2023):

*Probable CAPA:* Compatible clinical and imaging manifestations (pulmonary infiltrate or nodules preferably documented by chest CT or cavitating infiltrate), along with microbiological isolation of *Aspergillus* in BALF, galactomannan in BALF > 1, or galactomannan in serum > 0.5.

*Possible CAPA*: Compatible clinical and imaging manifestations, along with microbiological isolation of *Aspergillus* in respiratory specimens in sputum. Possible pulmonary CAPA requires pulmonary infiltrates, well-circumscribed lesions(s) or nodules, preferably documented by chest CT, or cavitating infiltrate that cannot be attributed to another cause. In patients with bilateral, ground-glass opacities or other COVID-19-related manifestations, significant radiological changes as described above and confirmed by a specialized radiologist are necessary to consider the possibility of CAPA.

The study was conducted in accordance with the ethical principles of Good Clinical Practice and the Declaration of Helsinki. The study was approved by the ethics committee of the First Affiliated Hospital of Zhengzhou University (Identifier: 2023-KY-0139-001). This study was registered in chictr.org/cn (registry number: ChiCTR2300070926).

## 3 Results

A total of 43 successive ARDS patients with COVID-19 (34 male and 9 female patients; median age, 60 years [IQR 44–76]) were detected. Of the 43 patients, 10 patients who tested positive for fungi via mNGS were included (**Table 1**).

**Table 1:** Characteristics of pulmonary critically ill patients with COVID-19 and positive for fungi by metagenomic analysis.

Characteristics	Patient #1	Patient #2	Patient #3	Patient #4	Patient #5	Patient #6	Patient #7	Patient #8	Patient #9	Patient #10
Demographics and clinical characteristics										
Gender	M	M	F	M	M	F	M	M	M	M
Age (years)	41	55	62	54	53	44	32	76	73	83
Diagnosis for COVID-19	PCR, mNGS	Antigen	PCR	PCR	Antigen	PCR, mNGS	PCR	PCR, mNGS	PCR	PCR, mNGS
Medical history	Hypertension, type 2 diabetes, kidney transplant status	Acute kidney injury, liver transplant status	Hypertension, nephritic syndrome	Hypertension, type 2 diabetes, liver abscess after percutaneous drainage	Thymoma B3	Hypertension, kidney transplant status	No	Coronary atherosclerotic heart disease after PCI, liver cirrhosis, hypersplenism, old cerebral infarction, type 2 diabetes, hypertension	No	Hypertension, coronary heart disease
Current smoker	Yes	No	No	Yes	No	No	Yes	No	No	No
Underlying immunocompromising condition	Steroids, tacrolimus, mycophenolate	Tacrolimus, mycophenolate, sirolimus	Mycophenolate	No	Oxaliplatin + cyclophosphamide + doxorubicine chemotherapy	Steroids, tacrolimus, mycophenolate	No	No	No	No
Cause of admission to the ICU	ARDS	ARDS	ARDS	ARDS, cerebral infarction, cerebral hernia, septic shock	ARDS	ARDS	ARDS, acute pancreatitis	ARDS	ARDS	ARDS, cardiopulmonary arrest after cardiopulmonary resuscitation
Time from illness onset to ICU admission, days	14	24	10	4	3	42	1	11	30	20
ARDS	Yes	Yes	Yes	Yes	Yes	Yes	Yes	Yes	Yes	Yes
PaO_2_ (ICU admission)	71	92	43	94	69	70	99	64	48	75
FIO_2_ (ICU admission)	0.90	0.95	0.61	0.41	0.7	0.60	0.41	0.81	1.00	0.80
Horowitz Index (ICU admission)	79	97	70	229	99	117	241	79	48	94
Prone positioning	Yes	Yes	Yes	No	No	No	Yes	Yes	No	No
Tracheal intubation	Yes	Yes	Yes	Yes	Yes	Yes	Yes	Yes	Yes	Yes
SOFA score (ICU admission)	7	8	4	7	6	7	4	7	9	8
CURB-65 score (ICU admission)	2	2	2	2	1	2	1	2	3	3
APACHE II score (ICU admission)	12	34	18	22	19	12	11	14	24	35
CAPA classification	Probable	Possible	Probable	Probable	Possible	Probable	Probable	Probable	Possible	Possible
Laboratory findings										
mNGS (first time after ICU admission)										
Specimen	BALF & PB	BALF & PB	BALF	BALF	BALF	BALF	BALF	BALF	BALF	BALF
Fungus	*Aspergillus fumigatus* (PB & BALF) *Aspergillus niger* (BALF) *Pneumocystis jeroveci* (BALF)	*Aspergillus flavus/Aspergillus oryzae*(BALF) *Aspergillus fumigatus* (PB)	*Aspergillus fumigatus* *Aspergillus flavus*	*Aspergillus fumigatus*	*Aspergillus fumigatus*	*Aspergillus flavus* *Aspergillus fumigatus*	*Aspergillus flavus/Aspergillus oryzae* *Rhizopus oryzae*	*Aspergillus flavus/Aspergillus oryzae* *Aspergillus fumigatus*	*Aspergillus flavus* *Aspergillus terreus* *Clavispora lusitaniae*	*Aspergillus fumigatus* *Candida albicans*
Bacteria	*Enterococcus faecium* (BALF) *Leuconostoc lactis* (BALF) *Tropheryma whipplei* (BALF)	*Klebsiella pneumoniae* (BALF & PB) *Acinetobacter baumannii* (BALF)	No	*Klebsiella pneumoniae* *Streptococcus pneumoniae* *Acinetobacter baumannii* *Enterococcus faecium*	*Acinetobacter baumannii*	*Enterococcus faecium* *Acinetobacter pittii*	*Escherichia coli* *Klebsiella pneumoniae*	*Acinetobacter baumannii*	*Klebsiella pneumoniae* *Enterococcus faecium*	No
Virology^1^	Epstein–Barr virus (PB) Human betaherpesvirus 5 (BALF) SARS-CoV-2 (BALF)	Human alphaherpesvirus 1 (PB) Epstein–Barr virus (PB)	No	Human alphaherpesvirus 1 Epstein–Barr virus	Human betaherpesvirus 5	Torque teno virus SARS-CoV-2	No	SARS-CoV-2	No	SARS-CoV-2
Serum galactomannan (µg/L) [<0.85]^2^	0.43	0.47	0.31	0.12	0.81	0.84	1.94	0.96	NA	0.28
Serum β-D-glucan (pg/mL) [0-95]	<10	<10	<10	171.11	<10	294.35	126.97	<10	NA	<10
BALF galactomannan (pg/mL) [<1]	0.48	0.36	NA	NA	NA	0.36	3.87	1.26	NA	NA
Sputum culture	Negative	*Acinetobacter baumannii*	NA	*Acinetobacter baumannii*	Negative	Negative	*Acinetobacter baumannii*	*Acinetobacter baumannii*	NA	NA
BALF culture	*Aspergillus flavus*	*Acinetobacter baumannii*	*Aspergillus fumigatus* *Aspergillus flavus*	Negative	Negative	Negative	*Escherichia coli* *Aspergillus flavus*	*Acinetobacter baumannii*	*Klebsiella pneumoniae*	Negative
Pheripheral blood culture	Negative	*Enterococcus faecium* *Klebsiella pneumoniae*	Negative	Negative	Negative	Negative	Negative	Negative	*Enterococcus faecium*	*Candida albicans*
Treatment										
Steroids to treat pneumonia	Yes	Yes	No	Yes	Yes	Yes	No	Yes	Yes	No
Antifungal	Voriconazole, Amphotericin B, Sulfamethoxazole	Voriconazole, Amphotericin B	Voriconazole	Voriconazole	Voriconazole	Voriconazole	Isavuconazole Amphotericin B	Voriconazole	No	No
Antiviral (besides COVID-19)	Penciclovir	Ganciclovir	No	No	No	Ganciclovir	No	No	No	No
Anti-COVID-19	Azvudine, Paxlovid	Paxlovid	Azvudine, Paxlovid	No	No	Paxlovid	No	Azvudine, Paxlovid	Azvudine, Paxlovid	No
Antibacterial	Meropenem, Linezolid	Ceftazidime and Avibactam Sodium, Polymyxin B	Ceftizoxime	Meropenem, Ceftazidime, and Avibactam Sodium	Imipenem and cilastatin sodium, Teicoplanin	Biapenem, Linezolid	Imipenem and Cilastatin Sodium, Ceftazidime and Avibactam Sodium, Teicoplanin	Moxalactam Cefoperazone Sodium and Sulbactam Sodium	Cefoperazone Sodium and Sulbactam Sodium	Imipenem and Cilastatin Sodium
Outcomes										
Acute renal failure	Yes	Yes	No	No	Yes	No	No	No	No	Yes
Renal replacement therapy	Yes	Yes	No	No	Yes	No	No	No	No	Yes
Vasopressor	Yes	Yes	No	Yes	Yes	No	Yes	No	No	Yes
ECMO	No	No	No	No	Yes(V-A)	No	Yes (V-V)	No	No	Yes (V-V)
Outcome	Died	Survived	Survived	Died	Died	Survived	Survived	Died	Died	Died

APACHE, Acute Physiology and Chronic Health Evaluation; ARDS, acute respiratory distress syndrome; BALF, bronchoalveolar lavage fluid; CAPA, COVID-19-associated pulmonary aspergillosis; CURB-65, Confusion, Urea, Respiratory rate, Blood pressure plus age ≥ 65 years; ECMO, extracorporeal membrane oxygenation; FIO_2_, fraction of inspiration oxygen; ICU, intensive care unit; mNGS, metagenomic next-generation sequencing; NA, not available; PaO_2_, partial pressure of oxygen; PB, peripheral blood; PCI, percutaneous coronary intervention; PCR, polymerase chain reaction; SOFA, Sequential Organ Failure Assessment; V-A, venoarterial; V-V, venovenous.

^1^In patient #2, #3, #4, #5, #7, and #9, only DNA sequencing was performed and no SARS-CoV-2 was detected.

^2^Normal reference range of our laboratory was given in square brackets. The same below.

**Figure 1:**
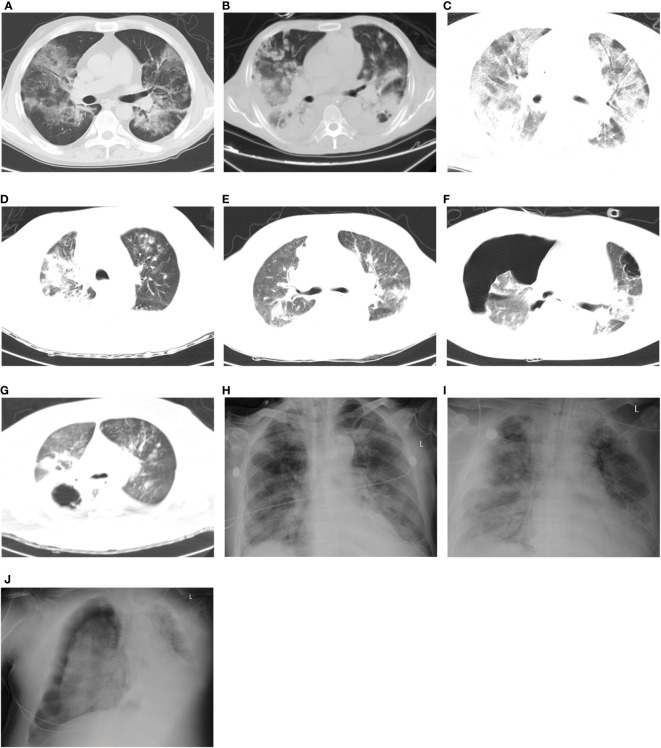
Thoracic CT-scan/x-ray of patients with COVID-19-associated pulmonary fungal infection. **(A)** Patient #1: Combined bilateral ground-glass opacities accompanied by mist-like shadows of high density. **(B)** Patient #2: Bilateral reductions in transparency with the presence of multiple nodular shadows of high density, accompanied by consolidations. **(C)** Patient #3: Bilateral multiple ground-glass opacities and patchy shadows. **(D)** Patient #4: The bronchovascular shadows bilaterally increased and blurred, accompanied by multiple patchy, cord-like, and round-like high-density shadows, as well as consolidations. **(E)** Patient #5: Bilateral ground-glass opacities present along with multiple patchy, cord-like high-density shadows. **(F)** Patient #6: Bilateral ground-glass opacities with patchy, cord-like high-density shadows, as well as cavities in both upper lung lobes. In addition, there was evidence of hydropneumothorax causing the right lung to collapse. **(G)** Patient #7: Bilateral ground-glass opacities with multiple small nodules and patchy, cord-like high-density shadows. Additionally, there were multiple cavities and consolidations observed in the right lung lobe. **(H)** Patient #8: Bilateral thickening of the lung texture and the presence of small exudation shadows. **(I)** Patient #9: Bilateral bronchovascular shadows that appeared increased and blurred, along with the presence of multiple exudations. **(J)** Patient #10: Bilateral increased thickening of the lung texture, with evidence of pleural effusion and atelectasis on the left side, as well as pneumothorax on the right side.

Patient #1: A 41-year-old man was admitted to the ICU because of severe ARDS with a Horowitz Index of 79 mm Hg. Nasopharyngeal swab PCR test showed that he was positive for SARS-CoV-2. He had a medical history of hypertension and type 2 diabetes. He underwent right kidney transplantation in 2003; tacrolimus, mycophenolate, and steroids were administered for immunosuppression therapy. Chest CT showed bilateral ground-glass opacities with mist-like high-density shadows (**Supplementary Video S1**; **Figure 1A**). PB was positive for *A. fumigatus*, and BALF was positive for *A. fumigatus, Aspergillus niger*, *Pneumocystis jeroveci, Enterococcus faecium, Leuconostoc lactis*, and *Tropheryma whipplei*, via the mNGS test. BALF culture tested positive for *A. flavus.* Intravenous voriconazole and inhalational amphotericin B were utilized for antifungal treatment. Sulfamethoxazole was administered as anti-pneumocystis prophylaxis. Meropenem and linezolid were adopted for antibacterial therapy. Penciclovir was adopted for antiviral therapy. Azvudine and Paxlovid were sequentially used for anti-COVID-19 therapy. Steroids were used to treat pneumonia, and prone positioning was also utilized. Because of acute renal failure, continuous renal replacement therapy (CRRT) was started.

Patient #2: A 55-year-old man was transferred to the ICU due to ARDS with a Horowitz Index of 97 mm Hg. Antigen rapid detection test revealed that he was SARS-CoV-2 positive (24 days before ICU admission). He had a medical history of acute kidney injury. He underwent liver transplantation owing to hepatitis B cirrhosis; tacrolimus and mycophenolate were administered for immunosuppression therapy. Chest CT showed bilateral decreased transparency and multiple nodular high-density shadows, with pulmonary consolidation (**Supplementary Video S2**; **Figure 1B**). *A. flavus*/*Aspergillus oryzae, Klebsiella pneumoniae*, and *Acinetobacter baumannii* were detected in BALF; *A. fumigatus, K. pneumoniae*, human alphaherpesvirus 1, and Epstein–Barr virus were detected in PB, via the mNGS test. *A. baumannii* was grown in sputum and BALF culture. *E. faecium* and *K. pneumoniae* were detected in PB culture. Intravenous voriconazole and inhalational amphotericin B were used for antifungal therapy. Ceftazidime, avibactam sodium, and polymyxin B were administered for antibacterial therapy. Penciclovir was used for antiviral therapy. Paxlovid was adopted to treat COVID-19. Steroids and prone positioning were also commenced. Owing to acute renal failure, CRRT was initiated.

Patient #3: A 62-year-old woman was transferred to the ICU by virtue of ARDS and having tested positive for SARS-CoV-2 via PCR. Her Horowitz Index was 70 mm Hg. Previous medical history suggested hypertension and nephritic syndrome. Mycophenolate was taken regularly for 2 years. Chest CT scan exhibited multiple ground-glass opacities and patchy shadows in both lungs (**Supplementary Video S3**; **Figure 1C**). *A. fumigatus* and *A. flavus* were both detected in BALF via mNGS and culture. Ceftizoxime was adopted for empirical treatment after ICU admission. Intravenous voriconazole was started after testing positive for *Aspergillus*. Azvudine and Paxlovid were successively commenced. Prone positioning was also put to use.

Patient #4: A 54-year-old man was admitted to the ICU because of mild ARDS (Horowitz Index of 229 mm Hg), coma, acute cerebral infarction, cerebral hernia, and septic shock. PCR test showed that he was positive for SARS-CoV-2. Previous medical history suggested hypertension, type 2 diabetes, and liver abscess after percutaneous drainage. Chest CT showed bilateral increased and blurred bronchovascular shadows with multiple patchy, cord-like, and round-like high-density shadows, as well as consolidations (**Supplementary Video S4**; **Figure 1D**). *A. fumigatus, K. pneumoniae*, *Streptococcus pneumoniae*, *A. baumannii, E. faecium*, human alphaherpesvirus 1, and Epstein–Barr virus were detected in BALF via mNGS analysis. Sputum culture grew *A. baumannii*, and drainage fluid of liver abscess culture grew *K. pneumoniae*. Intravenous voriconazole was commenced. Meropenem, ceftazidime, and avibactam sodium were utilized for antibacterial therapy.

Patient #5: A 53-year-old man presented with ARDS with a Horowitz Index of 99 mm Hg. Antigen rapid detection test suggested that he was SARS-CoV-2 positive. He was diagnosed with thymoma 20 days ago and was treated with oxaliplatin + cyclophosphamide + doxorubicine chemotherapy. Chest CT demonstrated bilateral ground-glass opacities and multiple patchy, cord-like high-density shadows (**Supplementary Video S5**; **Figure 1E**). *A. fumigatus, A. baumannii*, and human betaherpesvirus 5 were detected in BALF via the mNGS test. Imipenem, cilastatin sodium, and teicoplanin were adopted for antibacterial treatment. Intravenous voriconazole was started. On account of acute renal failure, CRRT was commenced. Owing to unsustainability of circulation, venoarterial (V-A) extracorporeal membrane oxygenation (ECMO) was put into use.

Patient #6: A 44-year-old woman was transferred to the ICU as a result of ARDS with a Horowitz Index of 117 mm Hg. PCR test suggested SARS-CoV-2 infection. She had a medical history of hypertension and uremia. She received kidney transplantation 10 years ago and was regularly taking tacrolimus and mycophenolate for antirejection therapy. Chest CT revealed ground-glass opacities with patchy, cord-like high-density shadows in both lungs, and cavity in the both upper lung lobes, as well as hydropneumothorax with a collapsed right lung (**Supplementary Video S6**; **Figure 1F**). *A. flavus, A. fumigatus, E. faecium, Acinetobacter pittii*, and Torque teno virus were detected in BALF via the mNGS test. Serum β-D-glucan also had a positive result. Intravenous voriconazole was utilized for antifungal therapy. Biapenem and linezolid were adopted for antibacterial therapy. Ganciclovir was used for antiviral therapy. Paxlovid was used for anti-COVID-19 treatment. Steroids were also started to treat pneumonia.

Patient #7: A 31-year-old man was admitted to the ICU because of mild ARDS (Horowitz Index: 79 mm Hg) and acute pancreatitis. PCR test showed that he was positive for SARS-CoV-2. Chest CT showed bilateral ground-glass opacities with multiple small nodules, patchy, cord-like high-density shadows, as well as multiple cavities and consolidations in the right lung lobe (**Supplementary Video S7**; **Figure 1G**). BALF was positive for *A. flavus*/*A. oryzae, Rhizopus oryzae, Escherichia coli*, and *K. pneumoniae*, via the mNGS test. *A. baumannii* was grown in sputum culture. *E. coli* and *A. flavus* were grown in BALF culture. Elevated galactomannan was seen in serum and BALF. Elevated serum β-D-glucan was also detected. Intravenous isavuconazole and inhalational amphotericin B were used for antifungal therapy. Imipenem, cilastatin sodium, ceftazidime, avibactam sodium, and teicoplanin were utilized for antibacterial treatment. Despite prone positioning, the patient required venovenous (V-V) ECMO supportive treatment to restore and maintain circulation stabilization.

Patient #8: A 76-year-old man was admitted to our ICU. He was intubated and developed severe ARDS with a Horowitz Index of 73 mm Hg. Nasopharyngeal swab PCR test showed that he was positive for SARS-CoV-2. He had a medical history of coronary atherosclerotic heart disease after percutaneous transluminal coronary intervention, liver cirrhosis, hypersplenism, old cerebral infarction, type 2 diabetes, and hypertension. X-ray radiography showed a combination of bilateral thickening of the lung texture and small exudation shadows (**Figure 1H**). The prehospital CT report suggested bilateral inferior pulmonary interstitial fibrosis and infection (no images available). *A. flavus*/*A. oryzae, A. fumigatus*, and *A. baumannii* were detected in BALF via the mNGS test. Elevated galactomannan in serum and BALF was detected. *A. baumannii* was detected in sputum and BALF culture. Intravenous voriconazole was used for antifungal treatment. Moxalactam or cefoperazone sodium and sulbactam sodium were used for antibacterial therapy. Azvudine or Paxlovid was separately adopted for anti-COVID-19 therapy. Steroids and prone positioning were also utilized. The patient eventually died of respiratory failure and respiratory cardiac arrest (Patient #1, **Table 1**).

Patient #9: A 73-year-old man presented with ARDS with a Horowitz Index of 48 mm Hg. PCR test revealed that he was SARS-CoV-2 positive. Chest x-ray imaged bilateral increased and blurred bronchovascular shadows, with multiple exudation (**Figure 1I**). Chest CT from the local hospital suggested bilateral ground-glass opacities (no images available). Azvudine and Paxlovid were already administered as anti-COVID-19 treatment at the local hospital. *A. flavus, Aspergillus terreus, Clavispora lusitaniae, K. pneumoniae*, and *E. faecium* were detected in BALF via mNGS. *K. pneumoniae* was grown in BALF and *E. faecium* was grown in PB. Cefoperazone sodium and sulbactam sodium were used for empirical treatment after ICU admission. The patient died of respiratory failure and malignant arrhythmias before the microbiological results became available.

Patient #10: An 83-year-old man was transferred to our ICU because of severe ARDS (Horowitz Index: 94 mm Hg) and cardiopulmonary arrest after cardiopulmonary resuscitation. PCR test suggested that he was SARS-CoV-2 positive. He had a medical history of hypertension and coronary heart disease. Chest x-ray radiography showed bilateral increased thickening of the lung texture, with pleural effusion and atelectasis on the left side, as well as pneumothorax on the right side (**Figure 1J**). BALF mNGS test revealed positive results for *A. fumigatus* and *C. albicans. C. albicans* was also detected in PB culture. V-V ECMO was performed after cardiopulmonary resuscitation. CRRT was adopted for renal replacement and toxin clearance. Imipenem and cilastatin sodium were utilized for empirical treatment after ICU admission. The patient died of respiratory failure and multiple organ dysfunction syndrome before antifungal therapy.

Among the 10 patients, 6 patients died, with all fatalities attributed to pulmonary co-infections caused by COVID-19 and subsequent multi-organ dysfunction, while the remaining 4 patients survived following treatment. Detailed patient characteristics are given in **Table 1**. Hematological test results for the 10 patients are presented in **Table 2**.

**Table 2:** Hematological test results for the 10 cases included in the study.

Hematological tests^1^	Patient #1	Patient #2	Patient #3	Patient #4	Patient #5	Patient #6	Patient #7	Patient #8	Patient #9	Patient #10
Blood routine examination										
WBC (10^9^/L) [3.5–9.5]^2^	14.79	6.16	18.79	16.51	8.32	14.42	29.72	3.39	10.83	23.35
HGB (g/L) [130–175]	87	73	142	121	117	109	141	131	112	92
PLT (10^9^/L) [125–350]	318	25	178	90	60	32	210	42	64	251
Lymphocyte ratio (%) [20–50]	1.9	4.8	1.8	6.7	9.9	0.5	4.2	3.5	1.5	8.7
Lymphocyte count (10^9^/L) [1.1–3.2]	0.28	0.3	0.34	1.11	0.82	0.07	1.25	0.12	0.16	2.03
PCT (ng/mL) [<0.064]	2.7	2.5	0.13	23	22	0.44	3.5	0.59	<0.07	31
CRP (mg/L) [<5]	59.16	165	60	119	152	25	450	74	30	89
IL-6 (pg/mL) [0–11.09]	10.46	585.05	1.5	21.01	175	436.58	133.26	7.87	111.48	321.84
BNP (pg/mL) [age-adjusted]^3^	5,110	20,500	1,930	248,000	161,000	1,450	735	1,565	797	7,930
Cr (μmol/L) [20–115]	315	244	66	73	238	55	52	60	107	101
Urea (mmol/L) [2.2–8.2]	19.33	24.12	9.9	10.38	17.2	20.68	7.63	10.4	19.71	12.77
PT (s) [8.8–13.6]	11	11.6	10.7	13.1	11.1	12.1	15.8	12.5	13.4	15.1
APTT (s) [26–40]	26.3	30.4	24.5	27.8	33.7	29.9	29	30	28.9	67.4
D-dimer (mg/L) DDU [0–0.55]	4.82	0.82	6.09	5.87	3.31	0.32	0.96	6.47	11.19	44.13
Glu (mmol/L) [3.6–6.1]	17.61	6.32	6.5	16.3	10.86	4.25	20.9	7.29	8.95	17.08
ALT (U/L) [0–40]	6	1	10	201	865	55	30	30	18	202
TBIL (μmol/L) [0–25]	10.4	8.6	13.3	16.6	10.6	28.3	29.3	18.5	7	14.6
Lac (mmol/L) [0.5–2.2]	1.1	1.1	1.6	1.5	2.6	1.2	1.8	1.7	1.2	15
TNT (ng/mL) [0–0.014]	0.064	0.066	0.018	0.1	9.53	0.06	0.013	0.015	NA	0
TNI (µg/L) [0.010–0.023]	0.019	0.02	<0.010	0.17	9.5	0.037	0.018	<0.010	0.021	0.062
CK-MB (ng/mL) [2–7.2]	4.2	<2	4.4	<2	395	10	3.01	3.8	<2	3.4
Lymphocyte analysis										
T (%) [50–81]	77.24	80.72	55.03	71.71	98.07	62.99	61.77	46.64	63.84	78.05
CD4+T (%) [27–51]	33.17	61.58	23.34	49.01	28.95	19.14	37.25	30.25	34.09	31.98
CD8+T (%) [15–44]	39.2	18.12	27.23	21.91	66.5	43.21	22.83	16.96	28.93	41.32
B (%) [5–8]	17.23	17.28	35.2	14.72	0	13.43	33.62	44.29	18.39	11.05
NK (%) [7–40]	12.5	1.36	9.49	12.53	1.56	50.41	3.17	5.48	11.47	5.86
CD4+T/CD8+T [0.71–2.78]	0.85	3.4	8.11	2.24	0.44	0.44	1.63	1.78	1.18	0.68
Lym (/µL) [1,530–3,700]	434.07	263	319.81	883	1,909	63.74	1,169.07	219	142.36	340.34
T (/µL) [955–2,860]	335.28	213	174.69	632	1,872	40.15	703.91	102	90.89	265.62
CD4+T (/µL) [550–1,440]	170.37	156	74.08	430	563	12.1	424.48	65	48.53	108.84
CD8+T (/µL) [320–1,250]	201.37	46	86.44	191	1,288	27.32	260.11	37	41.18	161.05
B (/µL) [90–560]	74.81	47	111.74	132	0	8.56	379.69	99	26.18	33.97
NK (/µL) [150–1,100]	12.28	4	30.13	112	29	11.8	36.16	12	16.32	18.02

ALT, alanine aminotransferase; APTT, activated partial thromboplastin time; ARDS, acute respiratory distress syndrome; B, B lymphocyte; BNP, N-terminal pro-brain natriuretic peptide; CK-MB, creatine kinase-MB; Cr, creatinine; CRP, C-reactive protein; Glu, glucose; HGB, hemoglobin; IL-6, interleukin-6; Lac, lactic acid; Lym, Lymphocyte; NA, not available; NK, natural killer cell; PCT, procalcitonin; PLT, platelet; PT, prothrombin time; T, T lymphocyte; TBIL, total bilirubin; TNI, high-sensitivity cardiac troponin I; TNT, high-sensitivity cardiac troponin T; WBC, white blood cell.

^1^The case sequence is identical to **Table 1**.

^2^Normal reference range of our laboratory was given in square brackets. The same below.

^3^Normal reference range of BNP in our laboratory: <300 pg/mL (<50 years); <900 pg/mL (50–75 years); <1,800 pg/mL (>75 years).

## 4 Discussion

Patients with ARDS induced by SARS-CoV-2 infection are prone to fungal infections despite having no previous immunodeficiency conditions (Buil et al., 2020; Koehler et al., 2020). Among 43 COVID-19 patients with moderate to severe ARDS with or without underlying immunocompromising factors on a single ICU, 10 were reported to be fungal positive using the mNGS test.

In the past few years, mNGS has been increasingly utilized in clinical diagnosis and has remarkable practical application value in the diagnosis of pathogenic microbial infection (Gu et al., 2019; Duan et al., 2021; Tang et al., 2021). In our study, the number of pathogenic microorganisms detected by mNGS was significantly higher than that by traditional methods, especially in the detection of fungi and viruses, although some studies report that there was no significant difference in the specificity between mNGS and conventional methods (Chen et al., 2020b; Huang et al., 2020b). The combined application of mNGS and traditional methods may provide robust backup in the diagnosis of pulmonary pathogenic microorganism infections. Metagenomic sequencing is a powerful tool in identifying newly emerging, uncommon, and unexpected pathogens. In certain circumstances, it can even be a life-saving test and the most dependable way to detect disease-causing microorganisms (Wilson et al., 2014).

For patients with pulmonary infections, PB and sputum specimens are more readily obtainable. In our center, traditional culturing and immunological testing remain the preferred diagnostic methods. However, the overall diagnostic efficiency is relatively low, often necessitating repeated testing to yield positive results or with consistently negative results, particularly in cases where empirical antibiotic treatment has been administered. BALF is highly sensitive for pulmonary infection detection and is the preferred specimen type, particularly in critically ill patients requiring fiberoptic bronchoscopy. BALF can be used for microbial culture, biochemical testing, cytological examination, parasitological examination, and mNGS testing. In this study, mNGS test results were obtained for the first time after ICU admission, and in most cases, BALF specimens were predominantly used. Therefore, the time of mNGS specimen submission typically lags behind that of conventional culturing and immunological testing.

Our objective is quite clear: we aim to utilize mNGS technology for early and rapid screening of potential pathogenic microorganisms, thereby providing specific guidance for further antimicrobial treatment. In the analysis of these 10 cases, it is evident that COVID-19 patients have a significantly high occurrence of concurrent infections with other pathogenic microorganisms, and there is also a high probability of detecting fungi. However, it is not possible to diagnose IFIs solely through mNGS testing, as positive fungal detection in BALF samples may also indicate colonization. The gold standard for IFI diagnosis is histopathological biopsy. Nevertheless, patient conditions often render this approach unrealistic, and as such, we did not pursue it. COVID-19 patients who develop IFI exhibit poor treatment responsiveness and high mortality rates (Baddley et al., 2021; Fortun et al., 2023). Consequently, when we detected fungi in BALF through mNGS, antifungal treatment for the patients was subsequently initiated. However, owing to the limited number of cases, it is challenging to determine whether this combined anti-infective therapy decisively contributed to the patients’ ultimate recovery. Given this limitation, it may be necessary to expand the sample size in future mNGS-related studies. Additionally, this highlights a shortcoming of mNGS, as it can detect a wide array of pathogenic species but cannot distinguish between pathogenic, colonizing, and contaminating microorganisms. Following pathogen detection, it is imperative to integrate other infection-related indicators in patients, such as blood PCT, CRP, BALF galactomannan, β-D-glucan, BALF cultures, and chest imaging, among others, to comprehensively evaluate the patient’s infection status and thereby guide treatment decisions. Ultimately, treatment effectiveness should be assessed based on the patient’s response to therapy.

There is a growing body of evidence suggesting that patients, especially those admitted to the ICU with COVID-19, may be at increased risk of developing fungal infections, such as aspergillosis, mucormycosis, candidiasis, and cryptococcosis (Amin et al., 2021; Doman and Banyai, 2022; Hoenigl et al., 2022). This association is likely due to several factors, including the immunocompromised effects of COVID-19, the use of corticosteroids and other immunomodulatory therapies in the treatment of severe COVID-19, and the use of invasive medical procedures such as mechanical ventilation, which can increase the risk of fungal colonization and infection (Baddley et al., 2021; Ezeokoli et al., 2021). *Aspergillus* infection was dominant in our study, as revealed by mNGS analysis indicating that all 10 patients were infected with or had co-infections involving *Aspergillus*. Likewise, some previous studies also documented a notable rise in the occurrence of aspergillosis among COVID-19 patients who were critically ill (Chen et al., 2020a; Koehler et al., 2020; Chong and Neu, 2021). It is important for clinicians to be aware of the increased risk of fungal infections in COVID-19 patients, particularly those who have underlying immunocompromising conditions, and to monitor for early signs of infection. Treatment of fungal infections in these patients may require aggressive antifungal therapy and invasive intervention in some cases. The results of mNGS and conventional diagnostic methods suggest that most of these patients also had concurrent bacterial or viral infections. Elevated inflammatory marks, such as white blood cell count, CRP, PCT, or IL-6, also suggest the possibility of concurrent infection. These patients progressed rapidly and had a poor prognosis; empirical use of antibiotics or antivirals before obtaining microbiological results may be reasonable.

The incidence of COVID-19-associated CAPA among patients who have been admitted to the ICU has been documented to vary from 19.6% to 33.3% (Fortun et al., 2023). Patients with CAPA had poorer prognoses, as evidenced by higher ordinal disease severity scores and longer recovery times, with mortality rates estimated to be approximately 50%, despite the widespread use of antifungals (Chong and Neu, 2021). In our study, of the 10 fungal-positive patients by mNGS testing, probable CAPA was diagnosed in 6 patients and possible CAPA was diagnosed in the remaining 4 patients. It is suggested that the results of mNGS testing and conventional diagnostic methods are largely consistent.

Lymphocyte analysis serves as a means to evaluate the immune system function, which includes the number and proportion of different types of lymphocytes in the blood (Luo et al., 2019). Lymphocyte analysis is of great significance for the diagnosis and prognosis evaluation of COVID-19 infection. COVID-19 infection may result in a decrease in lymphocyte count, especially CD8+ T, CD4+ T, and natural killer (NK) cells, which may lead to an impaired immune response to pathogens and aggravate the disease (Carsetti et al., 2020; Diao et al., 2020). Dysregulation of lymphocyte subsets may also be one of the reasons for the occurrence of dangerous and uncontrollable inflammatory response, namely, “cytokine storm”, in COVID-19 patients (Ghasemzadeh et al., 2022; Zanza et al., 2022). Some studies have found that the abnormal distribution of lymphocyte subpopulations may be associated with the severity of COVID-19 infection (Tan et al., 2020; Cantenys-Molina et al., 2021; Liu et al., 2021). We retrospectively reviewed the proportions and counts of T cells from the 10 patients (**Table 2**). Apart from one patient with thymoma (Patient #5), the T, CD4+ T, CD8+ T, and NK cell proportions in the other nine patients were mostly within the normal range or close to the normal range. However, the T, CD4+ T, CD8+ T, and NK cell counts of these patients were significantly decreased. These findings are consistent with literature reports, indicating that the immune system of patients with ARDS complicated by COVID-19 infection is significantly impaired. The lymphocyte ratio and count in blood routine examination also confirmed the immune system damage in these patients.

The SOFA scoring system has been widely applied in clinical research and critical care fields, especially in evaluating the condition and prognosis of patients with severe infections and ARDS. In our study, all 10 patients had a SOFA score greater than 2 upon admission to the ICU, indicating a high incidence of sepsis in patients with COVID-19 and ARDS. The CRB-65 score is used to assess the severity of community-acquired pneumonia and can help guide clinical decision-making for hospitalization and predict mortality. Interestingly, among the 10 patients, 8 patients had a CURB-65 score of less than 3 upon ICU admission. This suggests that for COVID-19 patients with ARDS, the CURB-65 score may need to be dynamically evaluated after admission. The Acute Physiology and Chronic Health Evaluation (APACHE) II score is a widely used clinical prediction tool that assesses the severity of critical illness in patients admitted to ICUs. Of the 10 patients, 6 patients had an APACHE II score greater than 15 upon ICU admission, indicating the severity of the disease.

In addition to respiratory symptoms, COVID-19 has been reported to cause damage to other organs, including the heart, liver, kidney, and the nervous system (Jain, 2020). Our study also supports the findings mentioned above. For example, some patients required renal replacement therapy or ECMO support (**Table 1**), while others exhibited abnormal coagulation, liver, kidney, or cardiac function indicators (**Table 2**). These extra-pulmonary manifestations highlight the systemic nature of COVID-19 and the need for comprehensive clinical management.

This study also has some limitations: (1) the sample size is relatively small, and (2) the data came from a single center during the outbreak of Omicron infection. Our findings need to be confirmed in clinical trials to elucidate the role of mNGS and COVID-19 in pulmonary critically ill patients in the future.

## 5 Conclusion

It is possible for COVID-19 patients to have a higher chance of developing fungal infections, along with simultaneous viral or bacterial infections. The utilization of mNGS can aid in detecting these infections effectively. Healthcare providers must be mindful of the heightened risk of fungal infections among COVID-19 patients, especially those with existing immunocompromised states. Early infection signs should be closely monitored.

## Data availability statement

The raw data supporting the conclusions of this article will be made available by the authors, without undue reservation.

## Ethics statement

The studies involving humans were approved by Ethic committee of the First Affiliated Hospital of Zhengzhou University. The studies were conducted in accordance with the local legislation and institutional requirements. The ethics committee/institutional review board waived the requirement of written informed consent for participation from the participants or the participants’ legal guardians/next of kin because (1) The research involves risks no greater than minimal risk, exclusively involves retrospective data collection, and does not interfere with clinical diagnosis and treatment; (2) Waiving informed consent will not adversely affect the rights or benefits of the subjects; (3) Patients have been discharged, and some have died. Without waiving informed consent, the research cannot proceed.

## Author contributions

Conceptualization, WG and MF; methodology, CH and SC; validation, RM, YS, and YL; data curation, YW; writing-original draft preparation, CH and RM; writing-review and editing, SC, YS, and YL; supervision, WG and MF. All authors contributed to the article and approved the submitted version.
